# Incidence, aetiology and outcomes of obstetric-related acute kidney injury in Malawi: a prospective observational study

**DOI:** 10.1186/s12882-018-0824-6

**Published:** 2018-02-02

**Authors:** William R. Cooke, Ulla K. Hemmilä, Alison L. Craik, Chimwemwe J. Mandula, Priscilla Mvula, Ausbert Msusa, Gavin Dreyer, Rhys Evans

**Affiliations:** 1 Department of Internal Medicine, Queen Elizabeth Central Hospital, Blantyre, Malawi; 2 Department of Obstetrics and Gynaecology, Royal Berkshire Hospital, Craven Road, Reading, RG1 5AN UK; 3 Department of Obstetrics and Gynaecology, Queen Elizabeth Central Hospital, Blantyre, Malawi; 4 University College London Centre for Nephrology, Royal Free Hospital, Pond Street, London, NW3 2QG UK; 5 Department of Nephrology, Bart’s Health NHS Trust, London, UK

**Keywords:** Acute Kidney Injury, Acute renal failure, Global Health, Sub-Saharan Africa, Pregnancy, Preeclampsia

## Abstract

**Background:**

Obstetric-related acute kidney injury (AKI) is thought to be a key contributor to the overall burden of AKI in low resource settings, causing significant and preventable morbidity and mortality. However, epidemiological data to corroborate these hypotheses is sparse. This prospective observational study aims to determine the incidence, aetiology and maternal-fetal outcomes of obstetric-related AKI in Malawi.

**Methods:**

Women greater than 20 weeks gestation or less than 6 weeks postpartum admitted to obstetric wards at a tertiary hospital in Blantyre, Malawi, and at high-risk of AKI were recruited between 21st September and 11th December 2015. All participants had serum creatinine tested at enrolment; those with creatinine above normal range (> 82 μmol/L) underwent serial measurement, investigations to determine cause of kidney injury, and were managed by obstetric and nephrology teams. AKI was diagnosed and staged by Kidney Disease Improving Global Outcomes (KDIGO) criteria. Primary outcomes were the incidence proportion and aetiology of AKI. Secondary outcomes were in-hospital maternal mortality, need for dialysis, renal recovery and length of stay; in-hospital perinatal mortality, gestational age at delivery, birthweight and Apgar score.

**Results:**

354 patients were identified at risk of AKI from the approximate 2300 deliveries that occurred during the study period. Three hundred twenty-two were enrolled and 26 (8.1%) had AKI (median age 27 years; HIV 3.9%). The most common primary causes of AKI were preeclampsia/eclampsia (*n* = 19, 73.1%), antepartum haemorrhage (*n* = 3, 11.5%), and sepsis (*n* = 3, 11.5%). There was an association between preeclampsia spectrum and AKI (12.2% AKI incidence in preeclampsia spectrum vs. 4.3% in other patients, *p* = 0.015). No women with AKI died or required dialysis and complete renal recovery occurred in 22 (84.6%) cases. The perinatal mortality rate across all high-risk admissions was 13.8%. AKI did not impact on maternal or fetal outcomes.

**Conclusions:**

The incidence of AKI in high-risk obstetric admissions in Malawi is 8.1% and preeclampsia was the commonest cause. With tertiary nephrology and obstetric care the majority of AKI resolved with no effect on maternal-fetal outcomes. Maternal-fetal outcomes in Sub-Saharan Africa may be improved with earlier detection of hypertensive disease in pregnancy.

**Electronic supplementary material:**

The online version of this article (10.1186/s12882-018-0824-6) contains supplementary material, which is available to authorized users.

## Background

Acute Kidney Injury (AKI) in pregnancy causes significant morbidity and mortality [1, 2]. It is increasingly recognised as a condition that is both preventable and treatable, often with simple and inexpensive interventions [3]. In developed countries, the incidence of AKI in pregnancy has been dramatically reduced as a result of improving clinical care [4]; a recent Canadian study found an AKI rate of 2.68 per 10,000 deliveries [5]. In developing countries, AKI in pregnancy is more common though less well studied, with AKI rates of 66 per 10,000 deliveries, for example, reported in Morocco [6].

The International Society of Nephrology recently launched the 0by25 initiative, which aims to eliminate preventable deaths from AKI by 2025 [7]. An initial goal is to establish the global burden of AKI, particularly in low-income settings. Over 50% of global maternal deaths occur in Sub-Saharan Africa (SSA), but the contribution of kidney disease to these is unknown [8]. Only three studies investigating AKI in pregnancy from SSA have been published since 1990; these report AKI limited to dialysis units, intensive care and women with severe preeclampsia [9–11]. In these studies, the predominant causes of AKI were preeclampsia-eclampsia, haemorrhage and sepsis.

To the best of our knowledge, there are no previous studies investigating obstetric AKI outside intensive care and dialysis units in SSA; the causes and consequences of AKI for mother and fetus are unknown. This study aimed to determine the incidence proportion, aetiology and maternal-fetal outcomes of AKI in obstetric patients at high-risk for AKI admitted to a tertiary hospital in Malawi. In a population with a high fertility rate (mean 5 births per woman in SSA [12]) and high prevalence of preeclampsia, sepsis and haemorrhage [13], we predicted a high rate of obstetric-related AKI, leading to adverse outcomes for mother and fetus.

## Methods

### Study setting and design

We conducted a prospective observational study between 21st September and 11th December 2015 in the Obstetrics and Gynaecology Department of Queen Elizabeth Central Hospital (QECH), Blantyre, Malawi. This unit delivers approximately 12,000 women per year and receives tertiary referrals from across the southern region of the country, although the majority of admissions are from within Blantyre district itself.

### Inclusion and exclusion criteria

All women aged 16 years and older, and greater than 20 weeks gestation or less than six weeks postpartum admitted to the Obstetric High Dependency Unit or high-risk areas of labour/antenatal/postnatal/gynaecology wards were assessed for risk of AKI within 48 h of admission. Presentation with one of 8 conditions was considered to place women at increased risk of AKI: gestational hypertension, preeclampsia, eclampsia, antepartum haemorrhage, postpartum haemorrhage, sepsis, heart failure and renal failure (Table 1). Patients with known renal failure were defined as a pre-existing diagnosis of CKD, or because a urea/creatinine result had already been obtained by the obstetric team and was elevated.

**Table 1:** Study inclusion criteria and definitions

Inclusion criterion	Definition
Gestational hypertension	New onset hypertension after 20 weeks gestation (defined as two BP readings > 140/90 separated by ≥4 h)
Preeclampsia	Gestational hypertension with dipstick proteinuria ≥1+
Eclampsia	Seizure with preeclampsia
Sepsis	Treating clinician’s judgement
Antepartum haemorrhage	Any documented vaginal bleeding except “spotting”
Postpartum haemorrhage	Any documented bleeding considered by the treating obstetrician not to be physiological
Heart failure	Pre-existing clinical diagnosis
Renal failure	Pre-existing clinical diagnosis

Definitions for hypertensive diseases from American College of Obstetricians and Gynecologists [23]

Study recruiters assessed whether AKI risk factor definitions were met; where insufficient information was documented to support or refute the presence of a risk factor, the treating obstetrician’s clinical diagnosis was used. All women at increased risk of AKI were enrolled in the study, baseline clinical data were recorded, and screening serum creatinine (SCr) was measured to determine the presence/absence of kidney disease. Screening SCr > 82 μmol/L was considered to be elevated, determined prospectively as being two standard deviations above mean SCr in the third trimester of pregnancy [14].

Women with elevated SCr on admission (> 82 μmol/L) were followed up with daily measurement of SCr and urine output, in addition to detailed clinical assessment and management by both the nephrology and obstetric teams. We recorded the nature and most likely causes of renal impairment based on the assessment of all available clinical and laboratory investigations as well as details of management thereafter. Patients with normal SCr (< 82 μmol/L) at enrolment were managed by obstetricians and not routinely seen by the study team. The development of AKI in hospital was not assessed.

Maternal and fetal outcomes, identified from ward discharge and delivery records as well as individual patient records, were recorded in all women. Hospital maternal mortality records were crosschecked to ensure that no women lost to follow-up had died. Women were followed up until death, discharge, or a predefined period of 2 weeks after last recruitment.

### Creatinine measurement

Serum creatinine (SCr) was tested (Jaffe method) using either a Flexor Junior Clinical Chemistry Analyser [Vital Scientific, The Netherlands] (31% samples) or Mindray Chemistry Analyzer BS-120 [Shenzen Mindray Bio-Medical Electronics Company, China] (69% samples). Biochemistry analysers were calibrated in accordance with the manufacturer’s instructions. A second analyser was used due to unresolvable technical problems with the original machine during part of the study.

### Definitions

AKI was diagnosed and staged according to Kidney Disease: Improving Global Outcomes (KDIGO) criteria [15] (Additional file 1). The lowest SCr recorded during admission or in the 3 months prior to admission was used as the baseline value. Imputed baselines based on a presumed normal Glomerular Filtration Rate (GFR) were not used in cases where the baseline was unknown.

Women with evidence of renal impairment (SCr > 82 μmol/L) which was thought to be of less than 3 months duration but who did not fulfil KDIGO AKI criteria were categorised as having an Acute Kidney Disorder (AKD) and were not included in any further analysis. Women with renal impairment of greater than 3 months duration were diagnosed with Chronic Kidney Disease (CKD) as per KDIGO and were not included in any further analysis [15]. AKI aetiology was determined, after detailed clinical review of each case, by a panel of clinicians led by a nephrologist (UH).

Complete renal recovery was defined as final measured SCr < 82 μmol/L after an initial SCr > 82 μmol/L. Where mothers remained in hospital solely because their child was receiving neonatal care or were transferred to private wards, maternal length of stay data were considered to be unrepresentative of maternal outcomes and were excluded from the analysis.

Perinatal mortality was defined as fetal death after 20 weeks gestation (stillbirth or termination of pregnancy) or neonatal death before discharge from hospital (early or late neonatal death). Preeclampsia spectrum was defined as gestational hypertension, preeclampsia or eclampsia.

### Outcomes

Primary outcome measures were the incidence proportion and aetiology of AKI. Secondary outcomes were maternal survival, need for maternal dialysis, renal recovery and maternal length of stay; fetal survival, gestational age at delivery, birthweight and Apgar score at 1 min after delivery [16].

### Statistical analyses

We conducted a descriptive analysis of the study cohort. Variables are reported as mean ± standard deviation (SD) or median ± inter-quartile range (IQR) depending on their distribution. We compared variables between women with NKD and any stage of AKI using the Student’s t test if normally distributed, the Mann-Whitney U test if not normally distributed and Chi-squared or Fisher’s exact test for proportions. Statistical analysis was performed using STATA version 10 (www.stata.com). A *p* value of < 0.05 was considered to represent statistical significance.

## Results

**Fig. 1:**
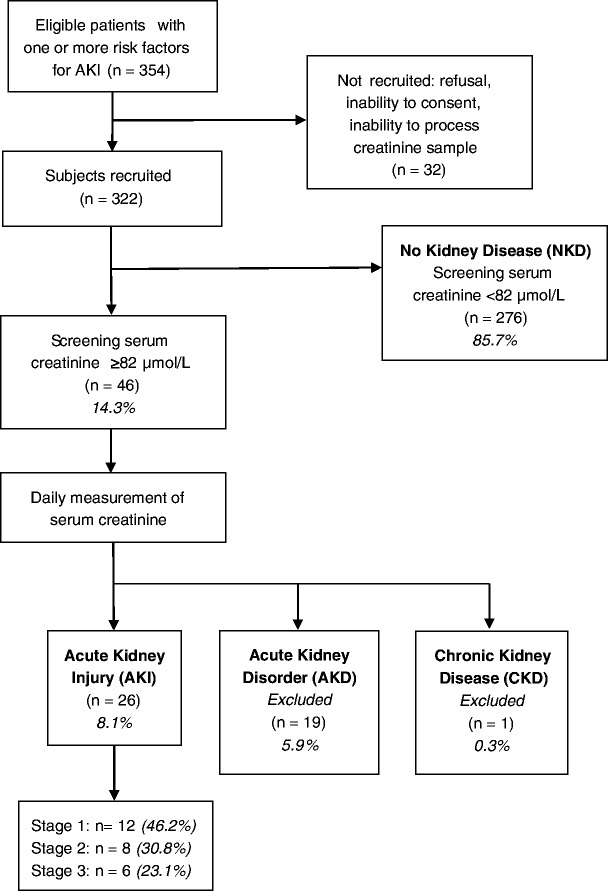
Flow chart of inclusion in the study

Study recruitment is summarised in Fig. 1. Two thousand two hundred forty-six women delivered during the study recruitment period.

### Baseline data

Demographic and baseline clinical data are reported in Table 2.

**Table 2:** Baseline data of women with acute kidney injury (AKI) and no kidney disease (NKD)

	NKD (*n* = 276)	AKI (*n* = 26)	*p* value
Age in years	25 (20–30)	27 (20–32)	0.44
Gravidity	2 (1–4)	2 (1–3)	0.43
Previous pregnancy loss	70 (25.4)	5 (19.2)	0.49
HIV positive	33 (12.0)	1 (3.9)	0.43
Antiretroviral therapy (prior to recruitment)	29 (10.5)	1 (3.9)	0.28
Diabetes as co-morbidity	1 (0.36)	0 (0)	0.76
Any nephrotoxins^a^ (prior to recruitment)	99 (36.0)	8 (30.8)	0.60

Figures shown are median (interquartile range) or frequency (%)

^a^Nephrotoxins = NSAIDs, tenofovir, gentamicin or traditional medications; recorded if taken within 1 week of recruitment

### Primary outcomes

#### Incidence of AKI

AKI of any stage was identified in 26 (8.1%) recruited women (Fig. 1). 12 (46.2%) cases of AKI were stage 1 [median screening SCr 99 μmol/L, IQR 94 – 118]; 8 (30.8%) cases were stage 2 [median screening SCr 99 μmol/L, IQR 96 – 120], and 6 (23.1%) cases were stage 3 [median screening SCr 139 μmol/L, IQR 135 – 148].

#### Aetiology of AKI

The primary causes of AKI were preeclampsia/eclampsia (*n* = 19, 73.1%), antepartum haemorrhage (*n* = 3, 11.5%), sepsis (*n* = 3, 11.5%) and cardiac failure (*n* = 1, 3.8%). The sources of sepsis were endometritis, chorioamnionitis and Bartholin’s abscess. In 13 (50%) cases, multiple factors contributed to development of AKI; nephrotoxins (NSAIDs, tenofovir, gentamicin or traditional medications) were a contributing factor in 10 (38.5%) cases.

### Secondary outcomes

Maternal outcomes were determined in 254 (84.1%) women. Two women died during their inpatient stay, neither had renal impairment. No woman required acute dialysis for AKI during her admission. Complete renal recovery occurred within 7 days in 22 (84.6%) AKI cases overall (75% of stage 1, 87.5% of stage 2 and 100% of stage 3 AKI). The remaining women (*n* = 4, 15.4%) were discharged before their creatinine fell below 82 μmol/L; three had evidence of partial renal recovery prior to discharge (discharge SCr was 84, 98, and 106 μmol/L); one had no renal recovery with discharge SCr 186 μmol/L.

Maternal length of stay was assessed in 234/302 (77.5%) cases: 16 mothers remained in hospital solely because their child was receiving neonatal care, 4 were transferred to private wards, 3 remained in hospital at the end of follow-up, and 45 were lost to follow-up. Median length of stay in women with NKD was 7 days (IQR 5 – 9) compared to 7 days (IQR 5 – 9) in women with AKI (*p* = 0.42).

Fetal outcomes were determined in 271/302 (89.7%) cases, 1 was lost to follow-up, and 30 were recruited antenatally and discharged prior to delivery. Fetal outcomes in patients with AKI and NKD are shown in Table 3. There were no statistically significant differences in any fetal outcome between AKI and NKD cohorts; median Apgar score at 1 min was lower in the AKI cohort (6) than in the NKD cohort (8), this approached statistical significance (*p* = 0.06).

**Table 3:** Fetal outcomes in patients with acute kidney injury (AKI) no kidney disease (NKD)

	NKD (*n* = 276)	AKI (*n* = 26)	*p* value
Gestational age in weeks (median; IQR)	37.7 (35.0–39.3)	37.6 (30.7–39.4)	0.56
Perinatal death (n; %)	33 (13.6)	4 (15.4)	0.8
Fetal weight in kg (median; IQR)	2.8 (2.2–3.3)	2.5 (1.6–3.3)	0.26
Apgar score (median; IQR)	8 (5.0–8.0)	6 (4.0–8.0)	0.06

Figures shown are median (interquartile range) or frequency (%)

Perinatal death occurred in 37/269 (13.8%) cases overall, regardless of renal impairment. One of the four perinatal deaths in AKI patients was a stillbirth at 30 weeks (in a mother with preeclampsia and APH, recruited at 3 days postpartum) suggesting fetal death likely preceded onset of AKI. The other three were early neonatal deaths: one mother with preeclampsia was recruited antenatally and delivered at 30 weeks; one delivered at 40 weeks and was recruited 2 days postpartum with endometritis; one delivered at 29 weeks with preeclampsia and sepsis and was recruited 2 days postpartum.

There was an association between preeclampsia spectrum (gestational hypertension, preeclampsia or eclampsia) and AKI. 20/164 (12.2%) women recruited with preeclampsia spectrum had AKI; 6/138 (4.3%) women without preeclampsia spectrum had AKI (*p* = 0.015).

## Discussion

### Main findings

To the best of our knowledge, this is the first study to prospectively investigate obstetric-related AKI outside intensive care and dialysis units in Sub-Saharan Africa. 8.1% of the cohort had AKI and the most common primary cause of AKI was preeclampsia spectrum, with a smaller contribution from sepsis and antepartum haemorrhage. No women with AKI died or required dialysis and the majority of AKI cases had complete renal recovery, even in stage 3 AKI. Fetal outcomes overall were poor with high rates of perinatal death. There were no statistically significant differences between AKI and NKD cohorts in either maternal or fetal outcomes.

We have identified ten publications investigating AKI in pregnancy from all of Africa since 1990 (Additional file 2). Three of these studies are from SSA; one investigates ICU patients [10], one investigates dialysis patients [9], and the other only includes patients with severe preeclampsia or eclampsia [11]. AKI prevalence amongst ICU patients varied from 6 to 34% and the commonest causes of AKI reported were preeclampsia-eclampsia, haemorrhage and sepsis.

A recent population-wide study from Canada reported an AKI incidence rate of 2.68 per 10,000 deliveries, with aetiologies similar to the previous African studies [5]. This study found AKI in 26 (8.1%) recruited women; given 2246 deliveries during the study period, we calculate the minimum incidence rate of AKI to be 116 per 10,000 deliveries in Malawi (over 40 times greater than the Canadian study). We have a novel cohort for SSA and cannot directly compare our AKI rate to that of previous ICU studies from the same region.

Whilst a recent review suggests sepsis and hypovolaemia are predominant causes of obstetric-related AKI in developing countries [1], our findings are consistent with those from other studies from Africa and match the patterns found in developed countries: preeclampsia-eclampsia underlies the majority of AKI. The association between preeclampsia spectrum at recruitment and AKI supports this. Table 4 demonstrates conditions contributing to the development of AKI in our cohort, as compared to data from Canada in 2007–10 [5]. Though the Canadian data define AKI based on clinician diagnosis as opposed to KDIGO criteria, there is an interesting comparison in AKI aetiology. Of note, there is a greater contribution of preeclampsia-eclampsia patients and a lesser contribution of postpartum haemorrhage to the development of AKI in our cohort.

**Table 4:** Conditions contributing to the development of AKI in this study, compared to National Canadian data for 2007–10 [5]

Condition	Malawi AKI cases (*n* = 26) Current study	Canadian AKI cases (*n* = 290) Mehrabadi et al. [5]
Antepartum haemorrhage	4 (15.4)	13 (4.5)
Postpartum haemorrhage	2 (7.7)	91 (31.4)
Gestational hypertension	2 (7.7)	37 (12.7)
Preeclampsia	14 (53.8)	120 (41.4)
Eclampsia	4 (15.4)	10 (3.5)
Sepsis	4 (15.4)	36 (12.4)
Cardiac failure	1 (3.8)	21 (7.2)

Multiple conditions are present in some patients. Canadian data from: Mehrabadi et al. [5]

Maternal outcomes in women with AKI were better than expected: no woman with AKI died or required dialysis. This may be accounted for by the quality of care in QECH, a tertiary centre with doctors providing care, regular monitoring, and some access to blood tests and medications. Renal impairment was detected early and women were managed jointly by nephrologists and obstetricians, a level of care which is not universal across SSA.

### Strengths and limitations

This study reports data from a new population: obstetric ward admissions at high-risk of AKI in SSA. It complements previous studies looking at ICU and dialysis patients, providing data on less apparently unwell patients. Malawi provides a unique context to perform this study in SSA. Free access to healthcare means the sample selected and the care provided for these women was not confounded by their ability to pay for care. Moreover, whilst QECH is a tertiary healthcare facility, it also acts as the district hospital for Blantyre and the majority of admissions represent provision of secondary care to the local population, making the study more generalisable for the country as a whole.

The study was performed in a challenging context where access to investigations is limited. Practical issues (e.g. lack of urine measuring jugs, lack of catheter bags with graduations to accurately measure urine volume) made collecting data on urine output difficult. We were unable (logistically and financially) to measure SCr in all patients who delivered due to the number of deliveries that occur at this centre, and some of these patients may have had kidney injury leading us to underestimate true incidence of AKI. Similarly, we could not undertake serial SCr in every recruited woman, so used a single screening SCr to identify patients at risk of AKI, which we then targeted with serial SCr measurement. We chose to use a threshold which is 2 standard deviations above the mean in the third trimester, which may not be representative of this African population: as stated, some patients with screening creatinine < 82 μmol/L may have had AKI, especially those with lower BMI. Nevertheless this is a lower threshold than other comparable studies from SSA (a study from Cameroon defined renal failure as creatinine > 97 μmol/L, a South African study as creatinine > 100 μmol/L [10, 11]) and highlights the challenges of diagnosing kidney disease in pregnancy more generally. Importantly, we only used this value to screen patients for kidney disease at recruitment, and AKI was confirmed on serial creatinine and urine output measurement thereafter and diagnosed according to the KDIGO criteria described. Serial SCr was not measured in women with a normal screening SCr and therefore hospital-acquired AKI was not assessed, although the majority of AKI in SSA is thought to be community acquired [17, 18].

Baseline data were recorded from the treating clinician’s notes, which may have been inaccurate. It was not possible to obtain urine dipstick results from all recruited patients. The small study size and possible underestimation of AKI may mean some true associations between AKI and maternal-fetal outcomes were not identified. The study was performed outside malaria season, one of the commonest causes of AKI in Malawi [18], and the pattern of AKI may be different at other times of the year.

Care provided to study participants at QECH might not be considered representative of care across SSA. The presence of an obstetrician-staffed Obstetric High Dependency Unit is uncommon in SSA. Patients in the study benefited from measurement of serum creatinine, a test not routinely performed at QECH in obstetric patients outside of research studies, and rarely available in any patient elsewhere in Malawi. Those with elevated creatinine benefitted from management from specialist renal services. As such, the study is likely to underestimate AKI severity and overestimate renal recovery.

### Interpretation

Overall, fetal outcomes were poor. The World Health Organisation reports the 2010 Malawi perinatal mortality rate to be 4% [19], which is significantly lower than the 13.8% in this study. Moreover, our narrower definition of perinatal mortality (only following neonates to discharge) likely underestimates the true value. Poor fetal outcomes might partly be accounted for by the recruitment of women with acute illness and preeclampsia itself may also play a role, given the majority of participants (*n* = 164; 54.3%) were recruited with this risk factor for AKI.

Preeclampsia is known to be a significant cause of obstetric AKI in developed and developing countries. A French ICU study reported 28% (24/85) of admissions with eclampsia, HELLP or preeclampsia without HELLP developed AKI [20]. A study from Cameroon found 18.5% (10/54) of admissions with severe preeclampsia and eclampsia had AKI on day-1 post-partum [11]. Our study supports these findings, reporting a significant association between preeclampsia spectrum and AKI; 12.2% women recruited with preeclampsia spectrum had AKI. We feel this is of relevance to settings across SSA where preeclamptic patients present to healthcare facilities that often lack the capability to diagnose AKI. Indeed, given the inclusion of less severe cases of preeclampsia in our study compared to those cited above, the burden of preeclampsia-related AKI is relatively high.

Reasons for the prevalence of preeclampsia and its associated poor outcomes include: late antenatal booking visits; infrequent antenatal care; limited access to routine antenatal ultrasound; minimal use of aspirin prophylaxis; and delivery of antenatal care by non medically trained health care workers. Poor access to diagnostics for and interpretation of blood pressure and proteinuria meant most preeclampsia was diagnosed when symptomatic, thus advanced and more severe in nature. Additionally, recent data demonstrate pre-pregnancy recovered AKI is a risk factor for subsequent preeclampsia and poor fetal outcomes [21]. In a country where AKI is common and severe [18], previous recovered AKI may be a significant contributor to the preeclampsia burden; the interaction of recovered AKI, preeclampsia, and poor fetal outcomes provides an important avenue of future study.

We feel improving diagnosis and treatment of preeclampsia is key to preventing AKI, and moreover, improving fetal outcomes in Malawi. In a multigravid population, preventing repeated cycles of preeclampsia-related AKI might in turn reduce the incidence of CKD. Better antenatal care is key to reducing the burden of preeclampsia and this will require increased provision of trained healthcare workers, better access to diagnostics including the development of simple point-of-care tests to detect kidney disease in rural settings [22], and population-level education regarding early presentation to antenatal care.

## Conclusions

The incidence of obstetric AKI in Malawi is significantly greater than in developed countries and this study provides novel and important data for obstetricians, nephrologists and governments in the provision of healthcare. The majority of AKI was caused by preeclampsia, a potentially detectable and treatable disease which is also likely to underlie much of the perinatal mortality identified. Poor fetal outcomes reflect the challenges of antenatal and perinatal care in Malawi. Future work should investigate obstetric AKI and preeclampsia in health centres and district hospitals, where facilities and staffing are poorer, and address whether improved recognition and treatment of preeclampsia can reduce the incidence of AKI and improve fetal outcomes in this part of the world.

## Additional files

Additional file 1: Definitions – Acute Kidney Injury, Acute Kidney Disorder, Chronic Kidney Disease, No Kidney Disease. Definitions from Kidney Disease: Improving Global Outcomes (KDIGO) criteria [15]. (DOCX 77 kb)

Additional file 2: Publications investigating AKI in pregnancy from Africa since 1990. Three studies from Sub-Saharan Africa are highlighted in grey. (DOCX 112 kb)

## Abbreviations

AKI: Acute Kidney Injury
CKD: Chronic Kidney Disease
NKD: No kidney disease
SCr: Serum creatinine
SSA: Sub-Saharan Africa
