# Supplementary material for: Incidence, aetiology and outcomes of obstetric-related acute kidney injury in Malawi: a prospective observational study
Source: BMC Nephrol. 2018 Feb 2;19:25. doi: 10.1186/s12882-018-0824-6 (PMC5797378; doi:10.1186/s12882-018-0824-6)
Supplement: Supplementary file 1 — Definitions – Acute Kidney Injury, Acute Kidney Disorder, Chronic Kidney Disease, No Kidney Disease. Definitions from Kidney Disease: Improving Global Outcomes (KDIGO) criteria [15]. (DOCX 77 kb) [file 12882_2018_824_MOESM1_ESM.docx]

| **Acute Kidney Injury (AKI)** | | |
| --- | --- | --- |
|  | Creatinine Criteria* | Urine Output criteria* |
| Stage 1 | 1·5-1·9 times baseline**/***  OR  ≥26·5μmol/l increase**** | <0·5ml/kg/h for 6-12 hours |
| Stage 2 | 2·0-2·9 times baseline | <0·5ml/kg/h for ≥12 hours |
| Stage 3 | 3·0 times baseline  OR  Increase in creatinine to ≥353·6μmol/l*****  OR  Initiation of RRT | < 0·3ml/kg/h for ≥24 hours  OR  Anuria for ≥12 hours |
| *basis of diagnosis can be with either creatinine criteria or urine output criteria, with most advanced stage on either determining maximum stage  **baseline creatinine = lowest creatinine within last year, or taken as lowest creatinine during hospital stay (whichever lower if multiple values)  ***known or assumed to have occurred within last 7 days  ****within 48 hours  *****must also fulfill at least stage 1 criteria | | |
| **Acute Kidney Disorder (AKD)** | | |
| Serum creatinine > 82μmol/l on admission but not fulfilling criteria for AKI during admission and no biochemical (previous creatinine) or structural (kidneys <9cm bilaterally) evidence kidney damage >3 months old | | |
| **Chronic Kidney Disease (CKD)** | | |
| GFR <60 ml/min per 1·73 m^2^ for >3 months* | | |
| *biochemical (previous creatinine >3 months old) or structural (kidneys <9cm bilaterally) kidney damage >3 months old | | |
| **No Kidney Disease (NKD)** | | |
| Serum creatinine < 82μmol/l on admission | | |
