# Supplementary material for: Incidence, aetiology and outcomes of obstetric-related acute kidney injury in Malawi: a prospective observational study
Source: BMC Nephrol. 2018 Feb 2;19:25. doi: 10.1186/s12882-018-0824-6 (PMC5797378; doi:10.1186/s12882-018-0824-6)
Supplement: Supplementary file 2 — Publications investigating AKI in pregnancy from Africa since 1990. Three studies from Sub-Saharan Africa are highlighted in grey. (DOCX 112 kb) [file 12882_2018_824_MOESM2_ESM.docx]

| **Study reference *Location*** | **Study design** | **AKI definition** | **Key results and conclusions** | **Haemodialysis available** |
| --- | --- | --- | --- | --- |
| Kabbali 2015  *Fez, Morocco*[1] | National prospective study over 6 months | RIFLE | 44 AKI patients; aetiologies: preeclampsia (64%), haemorrhagic shock (14%), sepsis (11%). 93% 3^rd^ trimester/postpartum. Maternal mortality 11.4% | Yes |
| Bentata 2015  *Oujda, Morocco*[2] | Retrospective study over 7 years in ICU patients >20 weeks gestation or <7 days postpartum | KDIGO | 186 patients included; 34% had AKI; causes: 59% preeclampsia, 36% haemorrhage, 31% sepsis. Maternal mortality 30%; perinatal mortality 59% | Yes |
| Kaze 2014  *Yaounde, Cameroon*[3] | Prospective study over 19 months in patients with severe preeclampsia/eclampsia >20 weeks gestation day 1 – 6 months postpartum | Serum creatinine  >1.1 mg/dL or creatinine clearance <90 ml/min | 54 patients; 13 (24.1%) and 10 (18.5%) patients had renal failure based on eGFR and serum creatinine respectively on day-1 post-partum. All AKI recovered within 3 months after delivery. | Yes |
| Kamal 2014  *Zagazig, Egypt*[4] | Prospective study over 3 years in ICU obstetric patients | RIFLE | AKI prevalence in ICU patients 5.9%; causes: HELLP (43%), pregnancy-related hypertension (30%), sepsis (10%). Maternal mortality 10% (31% of these due to AKI). | No |
| Bouaziz 2013  *Tunisia*[5] | Retrospective study over 17 years in ICU obstetric patients >20 weeks gestation | Serum creatinine  >8 mg/dL | 550 patients included; 57% had AKI; causes: preeclampsia (67%), acute haemorrhage (28%). Maternal mortality 6%. Mortality significantly higher in patients with PRAKI. | Yes |
| Arrayhani 2013  *Fez, Morocco*[6] | Prospective study over 1 year | RIFLE | 37 cases of PR-AKI; causes: preeclampsia (67%), haemorrhagic shock (25%) | Yes |
| Bentata 2012  *Oujda, Morocco*[7] | Retrospective study of ICU obstetric patients >20 weeks gestation | RIFLE | AKI prevalence in ICU patients 34%; causes: preeclampsia (61%), haemorrhagic shock (30%). Maternal mortality 28%; perinatal mortality 59% | Yes |
| Bentata 2011  *Oujda, Morocco*[8] | Study of women admitted to ICU >20 weeks gestation over 3 years | RIFLE | 43 cases | Yes |
| Drakeley 2002  *Cape Town, South Africa*[9] | Retrospective study of patients with renal failure, severe preeclampsia and oliguria admitted to obstetric ICU over 4 years. | Serum creatinine  ≥1.13 mg/dL | 89 cases; records available for 72. Perinatal mortality 38%; no maternal deaths. | Yes |
| Randeree 1995  *Durban, South Africa*[10] | Retrospective study of patients with PRAKI requiring dialysis over 3 years | Rising urea with falling urine output or fluid overload with urine output < 400 mL/day | 42 cases; causes: preeclampsia-eclampsia (commonest).  Maternal mortality 5%. | Yes |

**References**

1. Kabbali N, Mikou S, Bardai G El, Najdi A, Ezziani M, Zahra F. of Kidney Diseases and Transplantation Renal Data from the Arab World Eligibility for Renal Transplantation : A Moroccan Interregional Survey. 2015;26:153–60.

2. Bentata Y, Madani H, Berkhli H, Haddiya I, Saadi H, Mimouni A, et al. Acute kidney injury according to KDIGO stages and maternal mortality in the intensive care unit. Intensive Care Med. 2015;41:555–6.

3. Kaze FF, Njukeng FA, Kengne A-P, Ashuntantang G, Mbu R, Halle MP, et al. Post-partum trend in blood pressure levels, renal function and proteinuria in women with severe preeclampsia and eclampsia in Sub-Saharan Africa: a 6-months cohort study. BMC Pregnancy Childbirth. 2014;14:134.

4. Kamal EM, Behery MME, Sayed GAE, Abdulatif HK. RIFLE Classification and Mortality in Obstetric Patients Admitted to the Intensive Care Unit With Acute Kidney Injury: A 3-Year Prospective Study. Reprod. Sci. 2014;21:1281–7.

5. Bouaziz M, Chaari A, Turki O, Dammak H, Chelly H, Ammar R, et al. Acute renal failure and pregnancy: a seventeen-year experience of a Tunisian intensive care unit. Ren. Fail. 2013;35:1210–5.

6. Arrayhani M, El Youbi R, Sqalli T. Pregnancy-related acute kidney injury: experience of the nephrology unit at the university hospital of fez, morocco. ISRN Nephrol. 2013;2013:1–5.

7. Bentata Y, Housni B, Mimouni A, Azzouzi A, Abouqal R. Acute kidney injury related to pregnancy in developing countries: Etiology and risk factors in an intensive care unit. J. Nephrol. 2012;25:764–75.

8. Bentata Y, Housni B, Mimouni A, Abouqal R. Obstetric acute renal failure in an intensive care unit in Morocco. Int. J. Gynaecol. Obstet. Elsevier B.V.; 2011;115:196–8.

9. Drakeley AJ, Le Roux PA, Anthony J, Penny J. Acute renal failure complicating severe preeclampsia requiring admission to an obstetric intensive care unit. Am. J. Obstet. Gynecol. 2002;186:253–6.

10. Randeree IG, Czarnocki a, Moodley J, Seedat YK, Naiker IP. Acute renal failure in pregnancy in South Africa. Ren. Fail. 1995;17:147–53.
